# Automatic evaluation of the sinus of Valsalva from cine-MRI in patients with dilated aortic root

**DOI:** 10.1186/1532-429X-13-S1-P356

**Published:** 2011-02-02

**Authors:** Cédric Blanchard, Tadeusz Sliwa, Alain Lalande, Eric Steinmetz, Pauliah Mohan, Olivier Bouchot, Yvon Voisin

**Affiliations:** 1 LE2I UMR5158, Auxerre, France; 2 Service de Chirurgie Cardio-Vasculaire, CHU Dijon, Dijon, France

## Purpose

MRI appears to be particularly attractive for the study of the sinus of Valsalva (SV), however there is no global consensus on their measurements. The purpose of this study is to automatically evaluate the SV from cross-sectional cine-MR images and show that in this suitable plane diagnostic evaluation such as SV dilatation can be performed from the determination of the maximal radius.

## Methods

Cine-MR images were acquired using a breath-hold ECG-gated steady-state free-precession sequence (TR = 1.54 ms, TE = 1.49 ms, 17 lines per segment, α = 65°, slice thickness = 5 mm, temporal resolution = 27 msec/images, spatial resolution varied from 0.7 mm to 1.6 mm per pixel, according to the patient examination). The acquisition plane was oriented in aortic valve cross-section. Manual processing on the whole images remains a reliable but time-consuming solution. Then, an automatic method based on mathematical morphology, combining numerical geodesic reconstruction with RANSAC method to estimate area was elaborated in order to segment the aortic root. Relevant points, such as the commissures, the cusps and the centre of the SV are also automatically detected by using a distance transform for centre estimation and a radial study to find extremum radii points. Our method was tested on 13 examinations of patients with SV dilatation (2 women and 11 men, age: 54±11 years, body surface area: 1.97±0.19 m^2^). Considering all images, the maximum distance between two cusps determines the maximum radius. Manual measurement of this parameter was made by an experienced observer.

## Results

**Figure 1:**
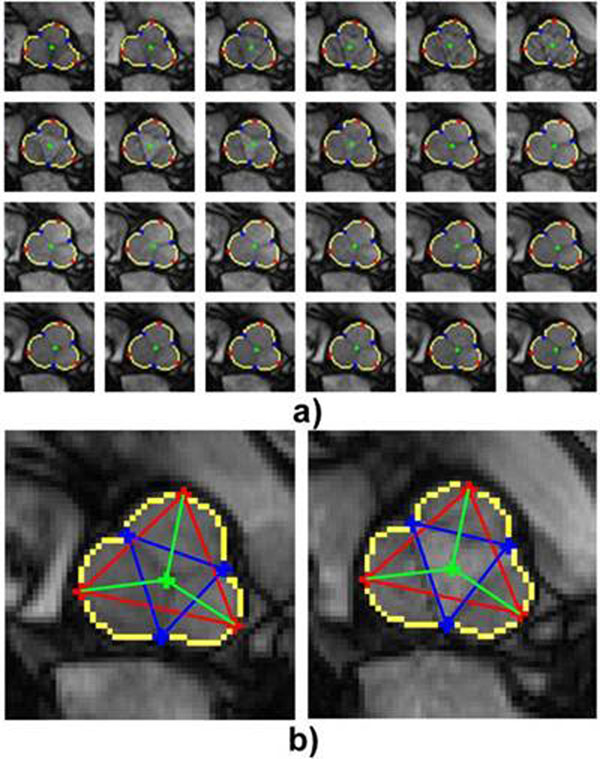
a) Segmentation on a whole image sequence with SV shape (yellow), centre (green), cups (red) and commissures (blue). b) Different measures on diastolic and systolic images.

There is an excellent correlation between manual and automatic measurements (r= 0.93; y = 0.93 x + 4.0; p<10^-3^). The corresponding Bland-Altman study shows an excellent concordance between the two approaches (mean of the differences = 0.5 mm and standard deviation of the differences = 2.3 mm). Figure 1

## Conclusions

Hence the cross-sectional orientation of the image acquisition plane combined with an automatic method provides reliable SV dilatation diagnosis, in particular by an automatic evaluation of the maximum diameter that is a crucial parameter in the follow-up of these patients. Moreover, the choice of this plane renders this evaluation robust in the case of the asymmetric dilatation and allows specifying the diagnosis because all distance between the cups, the commissures and the centre are available.

